# Beta-Amyloid Impairs Reelin Signaling

**DOI:** 10.1371/journal.pone.0072297

**Published:** 2013-08-12

**Authors:** Inmaculada Cuchillo-Ibáñez, Valeria Balmaceda, Arancha Botella-López, Alberto Rabano, Jesus Avila, Javier Sáez-Valero

**Affiliations:** 1 Instituto de Neurociencias de Alicante, Universidad Miguel Hernández-Consejo Superior de Investigaciones Científicas, Sant Joan d’Alacant, Alicante, Spain; 2 Centro de Investigación Biomédica en Red sobre Enfermedades Neurodegenerativas, Madrid, Spain; 3 Banco de Tejidos de la Fundación CIEN, CIEN Foundation, Carlos III Institute of Health, Alzheimer Center Reina Sofia Foundation, Madrid, Spain; 4 Centro de Biología Molecular "Severo Ochoa", Universidad Autónoma de Madrid, Consejo Superior de Investigaciones Científicas, Cantoblanco, Madrid, Spain; Universidad de Sevilla, Spain

## Abstract

Reelin is a signaling protein increasingly associated with the pathogenesis of Alzheimer’s disease that relevantly modulates tau phosphorylation. We have previously demonstrated that β-amyloid peptide (Aβ) alters reelin expression. We have now attempted to determine whether abnormal reelin triggered by Aβ will result in signaling malfunction, contributing to the pathogenic process. Here, we show that reelin forms induced by β-amyloid are less capable of down-regulating tau phosphorylation via disabled-1 and GSK3β kinase. We also demonstrate that the scaffold protein 14-3-3 that increases tau phosphorylation by modulating GSK3β activity, is up-regulated during defective reelin signaling. Binding of reelin to its receptor, mainly ApoER2 in the brain, relays the signal into the cell. We associate the impaired reelin signaling with inefficiency of reelin in forming active homodimers and decreased ability to bind efficiently to its receptor, ApoER2. More remarkably, reelin from Alzheimer cortex shows a tendency to form large complexes instead of homodimers, the active form for signaling. Our results suggest that reelin expression is altered by Aβ leading to impaired reelin signaling.

## Introduction

Increasing evidence suggests that Reelin expression is altered in the Alzheimer’s disease (AD) brain. Reelin is a signaling protein which modulates synaptic function and plasticity in the mature brain, and its signaling cascade can control tau phosphorylation [1,2]. The binding of Reelin to the transmembrane liporeceptors, apolipoprotein receptor 2 (ApoER2) or the very-low-density liporeceptor (VLDLR), relays the signal into the cell via the adapter Dab1 (disabled-1), initiating a cascade of intracytoplasmic events that ends with limited phosphorylation of the microtubule-associated tau protein, via inhibition of Glycogen synthase kinase 3 beta (GSK3β) activity; for a review, see [3].

Pathological hyperphosphorylation and aggregation of tau, concurrent with extracellular deposits of the β-amyloid protein (Aβ), are features of AD. Lack of Reelin is associated with increased tau phosphorylation [4], and mutations that prevent the Reelin-dependent induction of Dab1 tyrosine phosphorylation also cause tau hyperphosphorylation [5]. Reduced Reelin expression has been shown to accelerate tau pathology in transgenic AD mice [6]. Accordingly, Reelin depletion has been reported in affected brain areas of AD subjects and β-amyloid transgenic mouse models [7,8]. However, ours and other studies have demonstrated an increase in Reelin levels in the AD brain and in mice over-expressing Aβ [9–12]. This could be induced by β-amyloid as Aβ treatment elevates Reelin levels [11]. Increase in expression of the Reelin gene has been confirmed in two different AD cohorts [10,11], and has been associated with the specific vulnerability of neurons to AD [13]. We also have previously demonstrated that Aβ alters Reelin glycosylation, resulting in a glycosylation pattern similar to that of Reelin from cortex and cerebrospinal fluid (CSF) of AD patients [10,11]. The physiological consequences of alterations in Reelin expression are still unclear.

We have now attempted to determine whether abnormal Reelin triggered by Aβ treatment will result in signaling malfunction. In a cellular system, we study if an altered form of Reelin affects the Reelin signaling pathway, including Dab1, GSK3β and ultimately tau phosphorylation. We also illustrate that Aβ-altered Reelin glycoforms alters intracellular levels of the scaffold protein 14-3-3, a protein which promotes phosphorylated GSK3β to remain active, suggesting that this mechanism is also affected by impaired Reelin signaling. Finally, we investigated whether abnormal Reelin fails to bind ApoER2, the main brain receptor, and to form efficient signaling homodimers. We further examined the ability of Reelin species present in the AD brain to form homodimers. 

## Material and Methods

### Collection of human brain samples

This study was approved by the ethics committee of the Miguel Hernandez University and was carried out in accordance with the Declaration of Helsinki. Brain samples were obtained from the UIPA neurological tissue bank (Unidad de Investigación Proyecto Alzheimer, Madrid, Spain). After neuropathological examination, sporadic AD cases were categorized as stages V-VI of Braak and Braak (five cases; 66± 7 years). Samples from ND individuals (non-demented control subjects; five cases; 73± 2 years) corresponded to cases with no clinical dementia and no evidence of brain pathology. The mean postmortem interval of the tissue was 6 h, with no significant difference between each group of samples.

### Preparation of human brain samples

Samples (0.2 g) of human frontal cortex were homogenized (10% w/v) in 50 mM Tris-HCl, pH 7.4 / 150 mM NaCl/0.5% Triton X-100/0.5% Nonidet P-40 containing a cocktail of proteinase inhibitors (10,11). The homogenates were sonicated and centrifuged at 20,000×g at 4^°^ C for 20 min; the supernatant was collected and frozen at -80^°^ C. When samples were prepared for native-blue electrophoresis, the solubilization buffer contained 25 mM Bis-Tris pH 7.0, 0.5% n-dodecyl-beta D-maltoside (DDM), 20% glycerol.

### Cell cultures

SH-SY5Y cells were seeded in 75cm^2^ flasks at a density of 7×10^5^ cells/flask and cultured in DMEM supplemented with Glutamax (Invitrogen), 10% heat-inactivated fetal calf serum, 100 U/mL penicillin and 100 µg/mL streptomycin. To differentiate the cells, serum was reduced to 3% and 10 µM all-trans-retinoic acid (Sigma) was added for 5 consecutive days. Following this, serum was further reduced to 0.5% and 50 ng/ml of brain-derived neurotrophic factor (BDNF; Sigma) was added for the remaining culture period. Suspensions of β-amyloid 1-42 (Aβ42) or scrambled control peptide (Aβsc; AIAEGDSHVLKEGAYMEIFDVQGHVFGGKIFRVVDLGSHNVA) (both from American Peptide Company Inc.) dissolved in sterilized distilled water at a concentration of 1 mg/mL, were added one day after BDNF incubation at a final concentration of 10 µM, and this treatment was repeated 2 days later. The cell medium was no longer changed. Cells were cultured in presence of Aβ42 or Aβsc for 4 consecutive days. In some experiments, cells were treated with a lower dose of 1 µM Aβ42. Alternatively, cells were also treated for 24 h with 100 µM of deoxymannojirimycin (DMJ, Tocris) instead of Aβ.

SH-SY5Y cells cultured in 96-well plates and treated as previously described, were assessed for cell viability using the tetrazolium assay (MTS; CellTiter 96® AQueous Assay, Promega) according to the manufacturer’s instructions.

For primary cortical neuron cultures, cortical lobes from E16.5 mice embryos were trypsinized and dissociated in Hanks’ balanced salt solution (Life Technologies). Neurons were plated onto 35-mm dishes (1.3×10^6^ cells/dish) and maintained in Neurobasal medium (Invitrogen) containing B27 supplements (GIBCO BRL), 100 IU/ml penicillin, 100 µg/ml streptomycin and 2 mM glutamine. After 7 days, cortical neurons were treated with Reelin for 1 h. Cells were washed with Hank’s balanced salt solution (HBSS, Life Technologies) and scraped into 6× SDS-PAGE sample buffer, heated at 100^°^ C for 5 min and cell lysates stored at -80^°^ C.

### Reelin preparation

Medium from untransfected SH-SY5Y cells treated with (or without) Aβ was collected and centrifuged at 1,500×g for 5 min (this pellet was reserved for Western blotting analysis). The supernatant was filtered and concentrated using a Microcon filter with a 100 kDa cutoff (Millipore) to obtain enriched Reelin supernatants. Alternatively, in some cell media Aβ was removed by immunoprecipitation with the anti-Aβ polyclonal antibody 6E10 (Covance). Magnetic beads (PureProteome, Millipore) were coupled to 6E10 following manufacturer’s instructions. The 6E10-beads were incubated with medium from SH-SY5Y cells treated with (or without) Aβ for 4 h at 4^°^ C, then centrifuged at 500×g and the supernatant fraction re-incubated with fresh magnetic 6E10-beads at the same conditions. Immunocomplexes were separated by centrifugation and Aβ content was determined in the unbound fraction. These two successive incubations with the anti-Aβ beads ensured that most of the peptide in the samples was removed.

### Cell adhesion assay

Six-well plates were treated with nitrocellulose dissolved in methanol and left to dry. Then, wells were coated with equal amounts of Cont-Reelin or Aβ-Reelin, and left overnight at 37^°^ C. SH-SY5Y cells were plated and, after 2 h incubation, cells were washed extensively with Phosphate buffered saline. The remaining cells were counted. In some experiments Cont-Reelin was pre-incubated with the CR50 antibodies (20 µg/mL) for 1 h before coating.

### Western Blotting and antibodies

For Reelin detection brain extracts (30 µg) or SH-SY5Y supernatant samples (30 µl) were boiled for 3 min, and then resolved on 6% SDS-polyacrylamide gels (SDS-PAGE). Proteins were blotted onto nitrocellulose membranes and incubated with the anti-Reelin 142 antibody (1:500 dilution, Merck Millipore). Electrophoresis was allowed to proceed at a voltage that prevented excessive heat generation that affects Reelin detection [10,14]. As Reelin content varied broadly between different brain extracts, preliminary analysis was performed to ensure that the immunodetection signals lie within a linear range. For detection of other proteins, extracts from cultured neurons (15-25 µl) were boiled for 5 min in SDS-PAGE sample buffer. Levels of total tau (1:10000 dilution; DakoCytomation) and phospho-tau (AT8, Ser^202^, Innogenetics, at 1:2000 dilution; PHF13, Ser^396^, Abcam, at 1:2000 dilution) were detected by Western blot following resolution on 10% SDS-PAGE. Total GSK-3 (1:2000 dilution; Abcam), GSK3ser9 (1:2000 dilution; Abcam) and Dab1 (1:2000 dilution; Exalpha Biologicals, Inc) were resolved on 7.5% SDS-PAGE. PhosphoBlocker (5% from Cell Biolabs, Inc) was used to block membranes for phosphorylated proteins, and the 4G10 Platinum anti-phosphotyrosine antibody (1:2000 dilution; Merck Millipore) was used to detect phosphorylated tyrosines. The immunoreactivity of phosphorylated isoforms of Dab1, GSK-3 and tau were normalized to total levels of the respective protein; α-tubulin (Sigma-Aldrich) also served as a loading control. Aβ peptide from the supernatant of cells were analyzed by 10% Tris-tricine SDS-PAGE and detected with the anti-human Aβ antibody 4G8 (1:4000 dilution; Covance Research). For blue-native gel electrophoresis, samples were not boiled and electrophoresis was performed as previously described [15], using NativeMark™ Unstained Protein Standards (Life Techologies) as molecular weight marker. The signal was visualized by ECL (GE Healthcare Life Science) and analyzed using Science Lab Image Gauge v 3.0 software (Fujifilm).

The anti-Reelin CR50 antibody (MBL Int. Corp.) was used at 15µl/ml and incubated for 1 h with media containing Reelin to neutralize its function.

### Sucrose gradients

Reelin complexes were fractionated by ultracentrifugation at 150,000×g in a continuous sucrose gradient (5-20% w/v) for 18 hr at 4° C in a Beckman SW41 rotor. Brain extracts (~700 µl) were carefully loaded onto the top of the gradient containing 10 ml of 0.5 M NaCl, 50 mM MgCl_2_ and 0.5% (w/v) Triton X-100, in 50 mM Tris-HCl (pH 7.4). After centrifugation, the bottom of the tubes were punctured and 30-32 fractions were collected. Enzymes markers of known sedimentation coefficient (β-galactosidase, catalase and alkaline phosphatase) were used in the gradients to determine the approximate sedimentation coefficients.

### Lectin binding analysis of Reelin

Aliquots of brain extracts and SH-SY5Y cell medium were mixed with 40 µl of immobilized lectins Con A (lectin from *Canavalia* *ensiformis* ) and LCA (lectin from *Lens* *culinaris* *agglutinin*, both from Sigma-Aldrich) that recognize mannosyl residues, with each lectin having subtle differences in their structure. Con A binds mannose, while LCA also interacts with α-mannosyl residues of N-linked sugar chains while also requiring the presence of a fucose residue bound to the C-6 hydroxyl group of the GlcNAc at the reducing end for strong binding [16]. The Reelin-lectin mixture was incubated overnight at 4° C with gentle mixing; unbound Reelin was separated by centrifugation and examined by Western blotting.

### ApoER2-binding assay

Extracts from HEK-293 cells transfected with full-length mouse ApoER2 tagged with EGFP (gift from J. Nimpf) were incubated with an anti-GFP antibody immobilised in Sepharose (Abcam) for 4 h. Immobilized ApoER2-GFP was then incubated overnight with Reelin-enriched supernatants from Aβ-treated SH-SY5Y cells. Bound proteins were washed and both bound and unbound fractions were subject to Western blot analysis.

### Statistical analysis

Data are expressed as means ± SEM. Data were analyzed using SigmaStat (Version 2.0; SPSS Inc.) by Student’s t-test (two tailed) or by one-way analysis of variance (ANOVA), followed by Tukey test for pair-wise comparisons. Statistical significance was designated as *p* < 0.05. 

## Results

### Abnormal Aβ-Reelin fails to reduce tau phosphorylation

To assess the ability of altered Reelin expressed in presence of β-amyloid to activate its signaling cascade we used Reelin from an *in vitro* cell model. We have previously described that treatment of SH-SY5Y neuroblastoma cells with Aβ42 influences Reelin expression and induces changes in Reelin glycosylation that resemble Reelin glycoforms observed in AD brain [11]. Secreted Reelin was collected from untransfected SH-SY5Y cells treated without (Cont-Reelin) or with Aβ42 (Aβ-Reelin). Most of the soluble Aβ42 oligomers were removed during the centrifugation and posterior filtration process, and only traces of the peptide were resolved in fractions of the Reelin enriched supernatant, after SDS-PAGE and Western blotting (Figure S1A). Alternatively, Aβ was further depleted by two cycles of immunoprecipitation with the antibody 6E10; as a result of this, traces of Aβ were undetectable (Figure S1A). To avoid differences in the amount of Reelin obtained from SH-SY5Y-treated cells, the 420 kDa band from Cont-Reelin and Aβ-Reelin was quantified by Western blots and equalized. We first corroborated that secreted Cont-Reelin and Aβ-Reelin display differences in glycosylation and have different binding affinities to Con A and LCA, as we previously found in Reelin from human brain extracts [11]. Alteration in Reelin glycosylation was demonstrated by a lectin-binding assay based on lectins that recognize mannosyl residues, but which have subtle differences in their structure [16]. Western blot analysis of the Con A unbound-protein fraction indicated higher affinity for secreted Cont-Reelin (a low Reelin immunoreactivity in the unbound fraction indicates high affinity for the lectin) than for Aβ-Reelin, whereas Aβ-Reelin displays higher affinity to LCA (Figure S1B).

**Figure 1:**
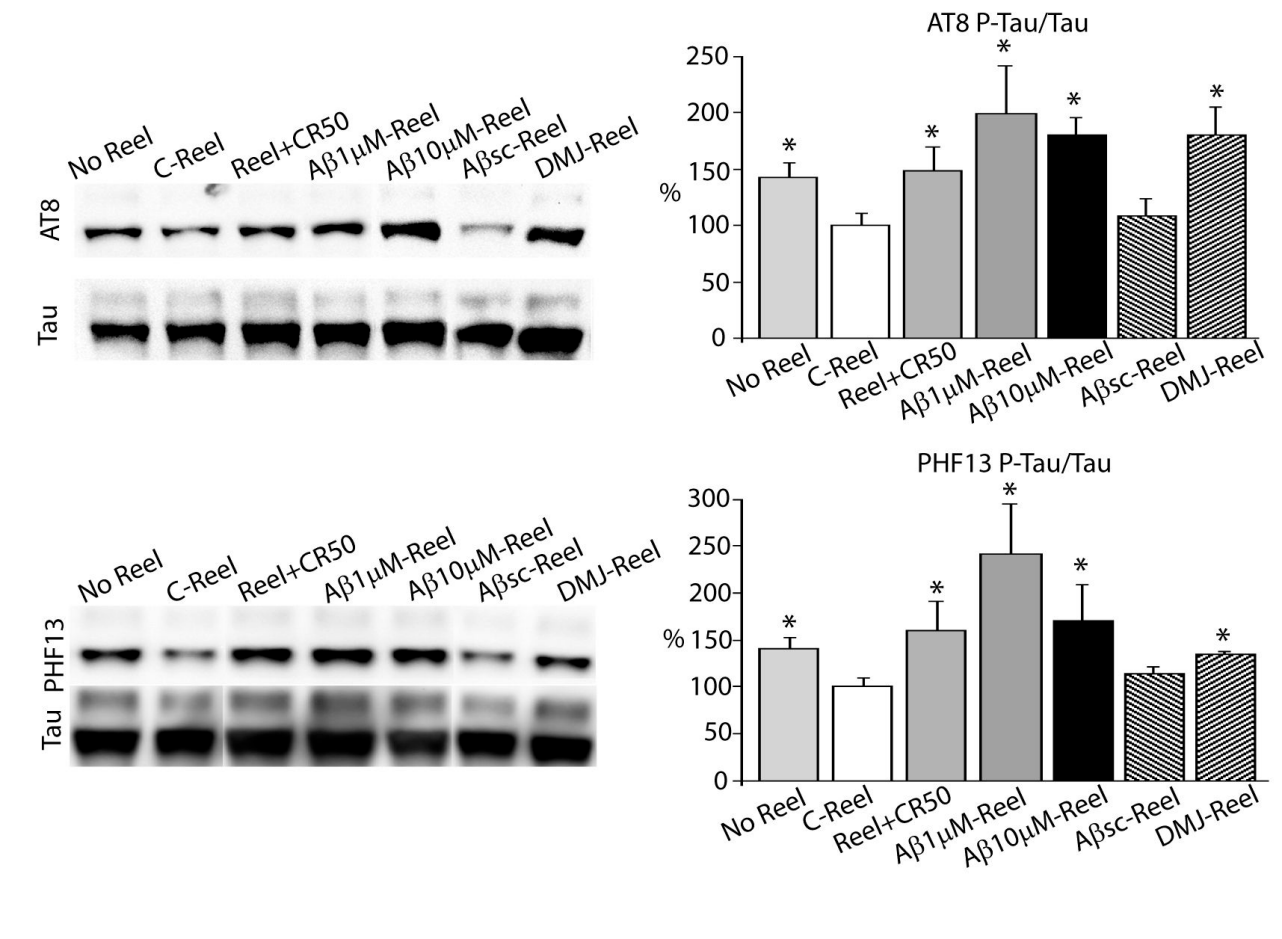
Aβ-Reelin fails to reduce tau phosphorylation. Levels of phospho-tau (P-tau; antibodies AT8 and PHF13) and total tau were calculated in primary mouse cortical neuron cultures treated with similar amounts of Reelin obtained from SH-SY5Y cells treated with 0 (control, C-Reel), 1 or 10 µM of Aβ42 (Aβ-Reel), or scrambled Aβ42 peptide (Aβsc-Reel; 10 µM) (at least n= 6, from 3 independent experiments). The comparison with non-treated cultures (No Reel) is also shown. In some experiments Cont-Reelin was pre-incubated with the antibody CR50 (15 µg/ml) for 1 h prior to treatment (n=3). Neuronal cultures were also incubated with Reelin glycoforms obtained from SH-SY5Y cells treated with 0 (control, C-Reel) or 100 µM of the mannosidase inhibitor DMJ (DMJ-Reel) (n= 6, from 2 independent experiments). The data represent the percentages of variation (means ± SEM) with respect to values determined for C-Reel treated cells. Data were analyzed using ANOVA analysis of variance, followed by Tukey test to compare all groups. *Significantly different (*p* < 0.05) from C-Reel treated cells.

Then, we determined if the changes in Reelin glycosylation by Aβ alter Reelin capacity to control tau phosphorylation. Mouse primary cortical cultures were treated with similar amounts of enriched Cont-Reelin or Aβ-Reelin preparations. Cont-Reelin decreases phosphorylation of tau, compared to non-treated neurons, whereas neurons treated with Aβ-Reelin leads to an increase in tau phosphorylation, estimated by the anti-phospho-tau antibodies AT8 (Ser^202^) and PHF13 (Ser^396^, an epitope phosphorylated by GSK3β, see 17), (Figure 1). Aβ-Reelin obtained from SH-SY5Y cells treated with 1 µM Aβ42 also fails to reduce tau phosphorylation. Moreover, Aβ-Reelin from SH-SY5Y cells treated with 10 µM Aβ42 and obtained after immunodepletion of the Aβ peptide, also increases tau phosphorylation (not shown). The changes in tau phosphorylation driven by treatment with Aβsc were similar to those detected in cells treated with Cont-Reelin. When neurons were treated with Reelin in the presence of the CR50, an anti-Reelin antibody which neutralizes Reelin [18,19], the phosphorylation levels of tau increases, confirming that the observed effects are Reelin dependent (Figure 1).

However, we cannot discard that undetectable traces of Aβ could affect directly tau phosphorylation in our cell system. To further confirm that changes in Reelin glycosylation can itself be responsible of impaired Reelin signaling, we employed also a different strategy to modify Reelin glycosylation. SH-SY5Y cells were treated with deoxymannojirimycin (DMJ), an inhibitor of endoplasmic reticulum mannosidases and Golgi mannosidase I. Neurons treated with altered Reelin glycoforms induced by DMJ also fail to reduce tau phosphorylation (Figure 1). These results confirm that altered Reelin glycosylation compromises its biological role of modulating tau phosphorylation.

**Figure 2:**
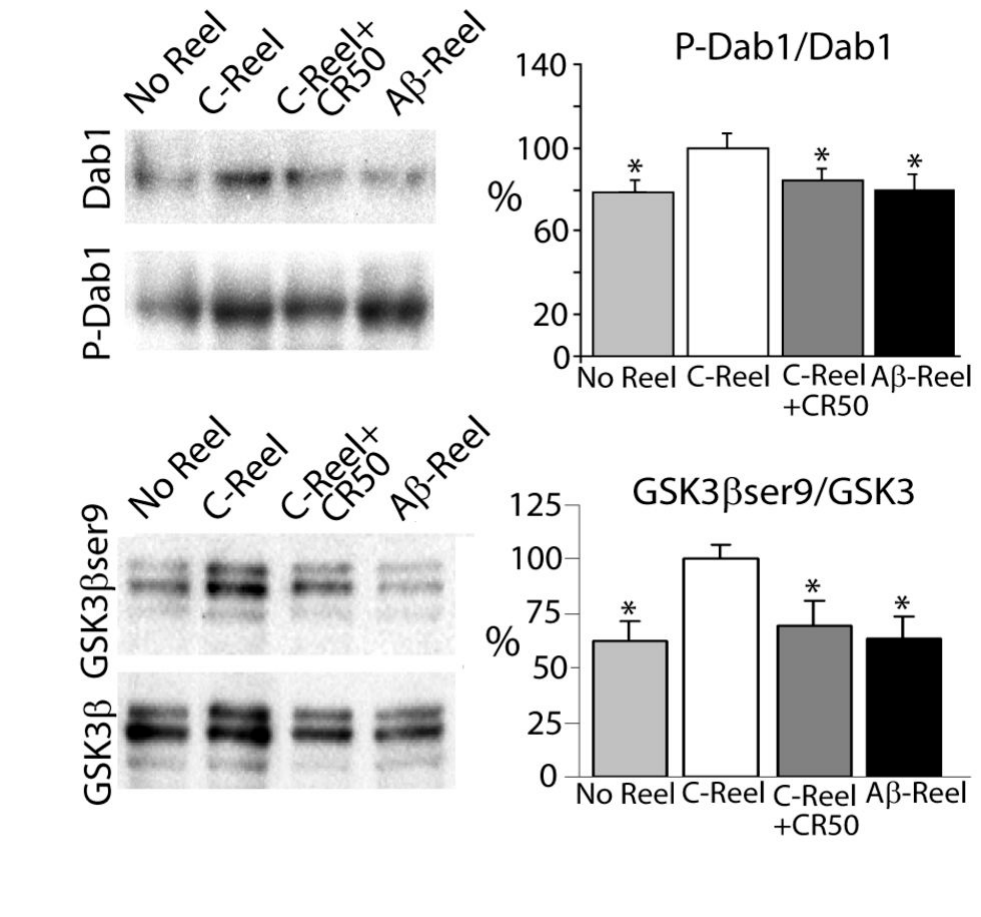
Aβ-Reelin fails to induce phosphorylation of Dab1 and GSK3β. Total cell lysates from primary mouse cortical neuron cultures treated without Reelin (No Reel) or with similar amounts of Reelin obtained from SH-SY5Y cells treated with 0 (control, C-Reel) or 10 µM of Aβ42 (Aβ-Reel) were probed with antibodies specific for total Dab1 and anti-phosphotyrosine Dab1 (P-Dab1), and GSK3β phosphorylated at serine 9 and total GSK3β. Levels of phosphorylated Dab1, GSK3β and tau were normalized to total Dab1, and GSK3β levels and tau respectively (n= 5 independent experiments for Dab1, GSK3β; n= 3 independent experiments for CR50). Data (means ± SEM) were analyzed using ANOVA analysis of variance, followed by Tukey test to compare all groups. *Significantly different (*p* < 0.05) from C-Reel treated cells.

Reelin initiates a cytosolic kinase pathway which includes phosphorylation of the Dab1 protein and phosphorylation of GSK3β at serine 9, which suppresses its activity, preventing hyperphosphorylation of tau [20]. Accordingly with the lack of modulation of tau phosphorylation, neurons treated with Aβ-Reelin show a decrease in phosphorylation of Dab1, as well as in phosphorylation of GSK3β at serine 9 (Figure 2).

### Involvement of the protein 14-3-3 in the Aβ-Reelin signaling

**Figure 3:**
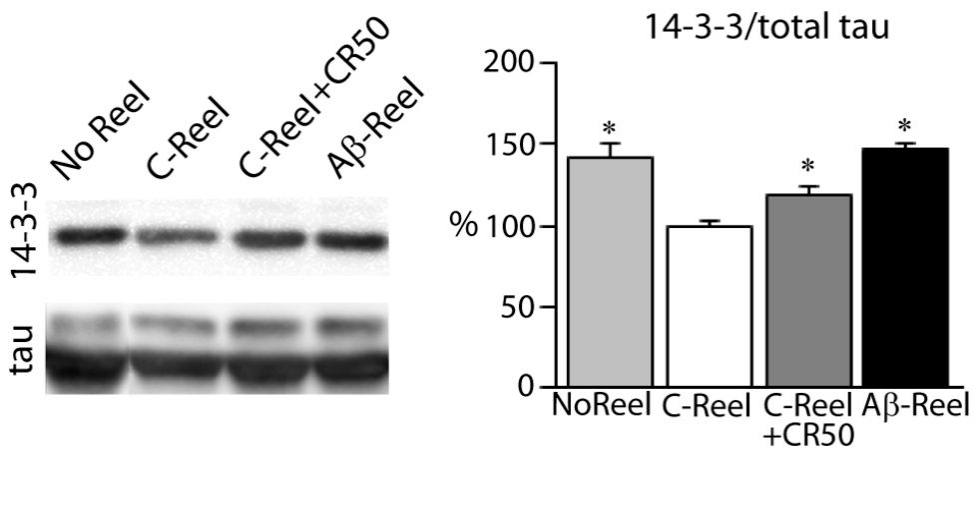
Increased 14-3-3 levels in neurons treated with Aβ-Reelin. Primary neurons were treated without Reelin (No Reel) or with Cont-Reelin (C-Reel) or Aβ-Reelin (Aβ-Reel), or Cont-Reelin pre-incubated with the antibody CR50 as described in Figure 1; and cell lysates were probed for 14-3-3 and normalized respect to total tau levels (n=3 independent experiments, values are means ± SEM). *Significantly different (*p* < 0.05) from C-Reel treated cells.

Tau and GSK3β form a complex where 14-3-3, a phospho-serine binding protein, may play a role by acting as bridge between these proteins. The 14-3-3 protein enhances tau phosphorylation by GSK3β favoring that GSK3β phosphorylated at serine 9 remains active and phosphorylates tau [21,22]. We have observed that the levels of 14-3-3 are significantly higher in cells treated with Aβ-Reelin compared to Cont-Reelin (Figure 3). Interestingly, neurons treated with Aβ-Reelin display similar 14-3-3 levels to those treated with the antibody CR50, which inhibits Reelin function. Our data indicate that 14-3-3 could be part of the Reelin signaling pathway, and suggests that this mechanism is also affected by the impaired Reelin signaling, and may contribute to the increment of tau phosphorylation.

### Aβ-induced Reelin species fail to form physiologically active dimers and bind less efficiently to ApoER2

**Figure 4:**
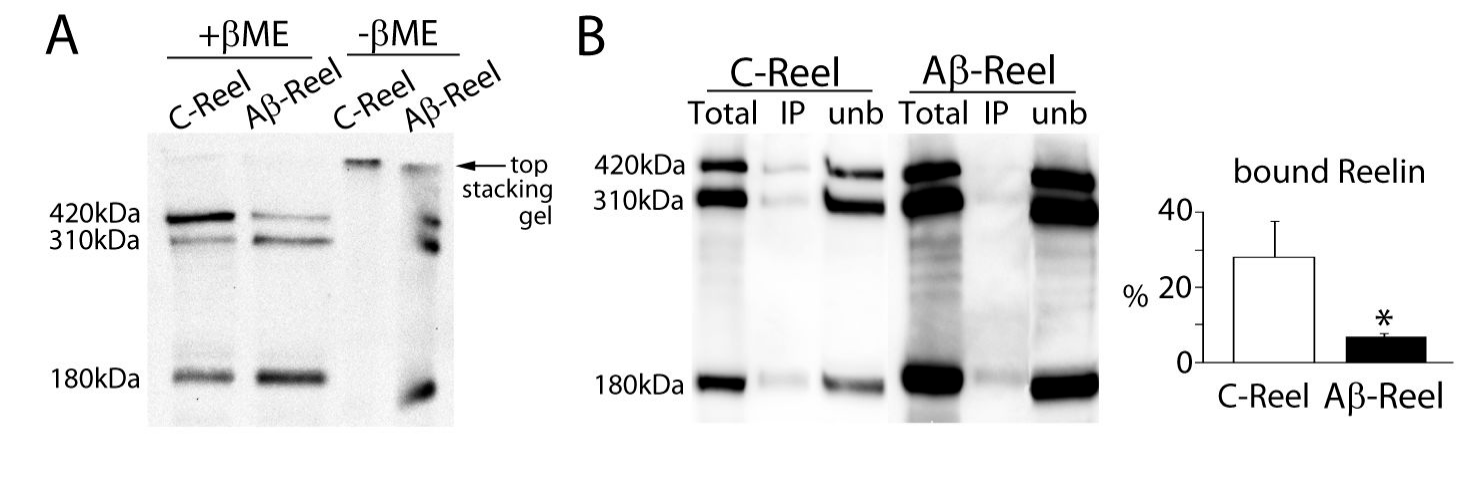
β-Amyloid compromises Reelin dimerization and affinity for ApoER2. (**A**) Aβ-induced Reelin cannot form covalent homodimers. Cont-Reelin or Aβ-Reelin glycoforms were denatured in the presence or absence of SDS/β-mercaptoethanol (βME) and subjected to SDS-PAGE followed by immunoblotting with anti-Reelin antibody (representative blots from 4 different experiments are shown). (**B**) Immobilized ApoER2 was incubated with enriched soluble-Reelin supernatant obtained from untransfected SH-SY5Y cells treated with 0 (control, C-Reel) or 10 µM Reelin (Aβ-Reel), and the precipitated protein (IP or bound) fractions were assessed by immunoblotting with an anti-Reelin antibody. The average percentage of the 420-kDa Reelin binding to immobilized ApoER2, expressed as mean± SEM of 4 independent determinations, is shown. Reelin which did not bind to ApoER2 (unb) is also shown. t-Test; *p< 0.05.

Reelin dimer formation is required for the full biological activity of the protein and to transduce its signaling [23,24]. Secreted disulfide-linked homodimer of Reelin are the forms found *in vivo* in brain and plasma [14,25,26]. Non-covalent (mainly electrostatic) interactions, also participate in the formation of functional Reelin dimers; but large complexes based only in electrostatic interactions fail to transduce signaling [24,25]. Thus, we next analyzed if the ability of Reelin to dimerize is affected when the protein is expressed in presence of Aβ. Supernatants containing Cont-Reelin or Aβ-Reelin were prepared for Western blotting in the presence and absence of SDS and β-mercaptoethanol in the sample buffer during the denaturation step, and then, both the stacking and resolving gel were examined. When disulfide bonds were not reduced Cont-Reelin failed to enter the SDS-PAGE resolving gel and most material remained at the top of the stacking gel; while Aβ-Reelin was resolved as monomer bands of 420, 310 and 180 kDa (Figure 4A), indicating that Reelin present in AD brain may not have capacity to form covalent dimers. Previous studies have also used a cell adhesion assay to demonstrate Reelin homophilic interaction [25]. In our experiments, cells plated in dishes coated previously with Aβ-Reelin attach in a lesser number than when dishes are coated with Cont-Reelin, indicating an impaired homophilic interaction for abnormal Reelin (Figure S2). Altogether, these results indicate that covalent dimerization is impaired in Aβ-Reelin.

We also examined if failure of Reelin to form dimers affects its capacity to bind ApoER2. This Reelin-ApoER2 interaction was examined by an ApoER2 binding assay using recombinant ApoER2. While both Aβ-Reelin and Cont-Reelin were able to bind recombinant ApoER2, Aβ-Reelin had decreased binding capacity (Figure 4B).

### Reelin from AD brain fails to form physiologically active dimers

**Figure 5:**
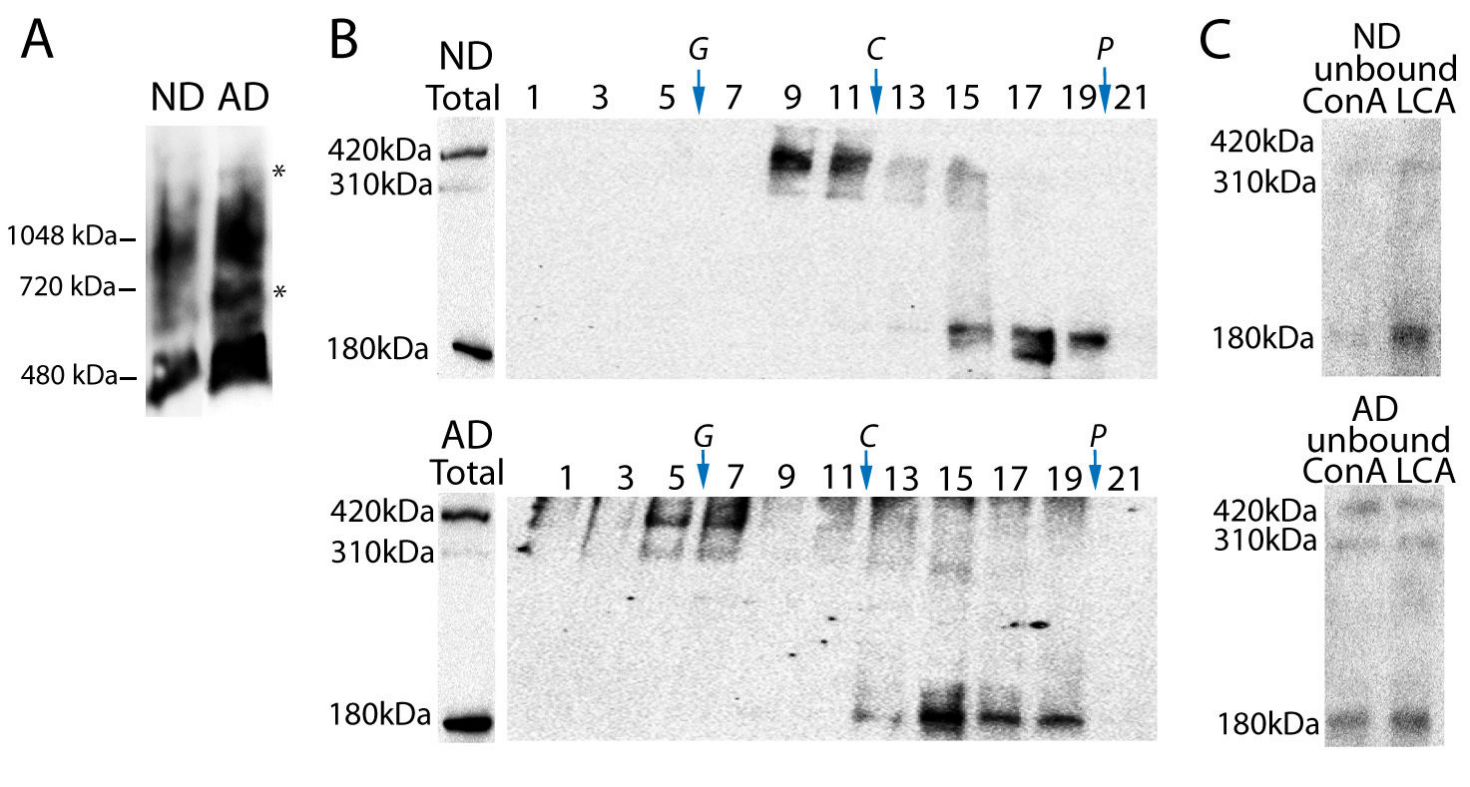
Reelin from AD cortex form large complexes. (**A**) Brain Reelin complexes from frontal cortex of ND and AD subjects (extracted in buffer containing 0.5% DDM) were analyzed by blue native-PAGE. * Asterisks indicate presence of intermediate and large Reelin complexes in AD samples. (**B**) Reelin extracted from frontal cortex of the same ND and AD subjects in presence of Triton X-100/Nonidet P-40 were applied to 5-20% sucrose density gradients (fractions were collected from the bottom of each tube). Aliquots from odd numbered fractions were immunoblotted for Reelin under denaturing conditions with the anti N-terminal Reelin antibody 142, which allows identification of the full-length 420 kDa Reelin and the two N-terminal 310 and 180-kDa fragments. Enzymes of known sedimentation coefficient, β-galactosidase (G, 16.0S; MW~ 540 kDa), catalase (C, 11.4S; MW~ 232 kDa) and alkaline phosphatase (P, 6.1S; MW ~ 140-160 kDa) were used as internal markers. (**C**) The same brain extracts were analyzed by lectin binding using Con A and LCA. The unbound Reelin glycoforms were determined by Western blotting (right panels). Representative cases from five independent experiments are shown.

While several studies have determined Reelin levels in AD brain and CSF, Reelin functionality has not been determined. Therefore, we examined Reelin isolated from the frontal cortex of AD subjects by blue native-PAGE to determine if these form homodimers or instead form larger inactive complexes. Complexes were extracted in assay buffer containing 0.5% DDM [27]. In ND (non-demented) brain extracts, as expected, a band was identified at ~450 kDa, representing the full-length protein, together with a high molecular mass band of ~800 kDa, probably corresponding to dimers of Reelin (Figure 5A). However, in the AD brain Reelin complexes appeared from ~450 to ~1400 kDa, with several Reelin immunoreactive bands corresponding to intermediates and large complexes (Figure 5A).

The Reelin complexes were further characterized by sedimentation analysis. Triton X-100/Nonidet P-40 extracts from the frontal cortex of AD and ND subjects were also fractionated on sucrose density gradients and resolved by Western blotted under denaturing conditions in order to characterize the full-length 420 kDa Reelin and the two N-terminal 310 and 180-kDa fragments. Reelin from AD extracts sediments in denser fractions than Reelin from ND extracts (Figure 5B). In the AD brain, Reelin forms complexes of higher molecular mass than the functional homodimers present in the non-pathological condition, and this could lead to an impaired capacity to bind correctly to its receptor. We have previously demonstrated altered Reelin expression as the result of an altered glycosylation pattern of the protein in the AD frontal cortex [10,11]. We corroborated that Reelin glycoforms extracted from ND brain cortex bind strongly to Con A than to LCA (decreased Reelin immunoreactivity); whereas in AD samples Reelin glycoforms have similar or higher affinity for LCA compared to Con A (Figure 5C). 

## Discussion

Levels of Reelin in AD cortex have been analyzed due to its important role in modulation of synaptic transmission and tau hyperphosphorylation, both significantly altered in this disease. An efficient Reelin signaling cascade controls tau phosphorylation, thus a reduction in Reelin may favor progression of the disease. However, Aβ treatment elevates Reelin levels and alters its glycosylation [11]. The physiopathological significance of an increased expression of altered species of Reelin in affected brain regions from AD patients remains unclear; and evaluation of its levels by Western blotting or immunohistochemistry alone, will not determine the correct function of the protein. Our present results indicate that Aβ compromises Reelin biological function by altering binding affinities to its receptor. Therefore, in spite of potential increased levels of the protein, this Reelin should result nonfunctional and Reelin signaling ends impaired in the AD brain.

Our data indicate that Reelin present in AD brain it is not capable of forming active homodimers. The possibility that Reelin binds to itself was proposed [25], and demonstrated *in vivo* [14,26]. Indeed, covalent homodimers of Reelin are the functional units which interact with receptors [23]. The covalent bond could be via a unique cysteine residue in the central region of Reelin [24]. N-terminal Reelin fragments form large but unstable assemblies that do not induce efficient phosphorylation of Dab1 [26]. The sedimentation analysis demonstrates differences between Reelin species extracted from AD and ND brains. Changes in glycosylation characterized in Reelin extracted from AD brain may result in abnormal homophilic interaction of altered Reelin glycoforms forming inactive high molecular mass complexes. Changes in glycosylation may also affect Reelin/ApoER2 interactions.

**Figure 6:**
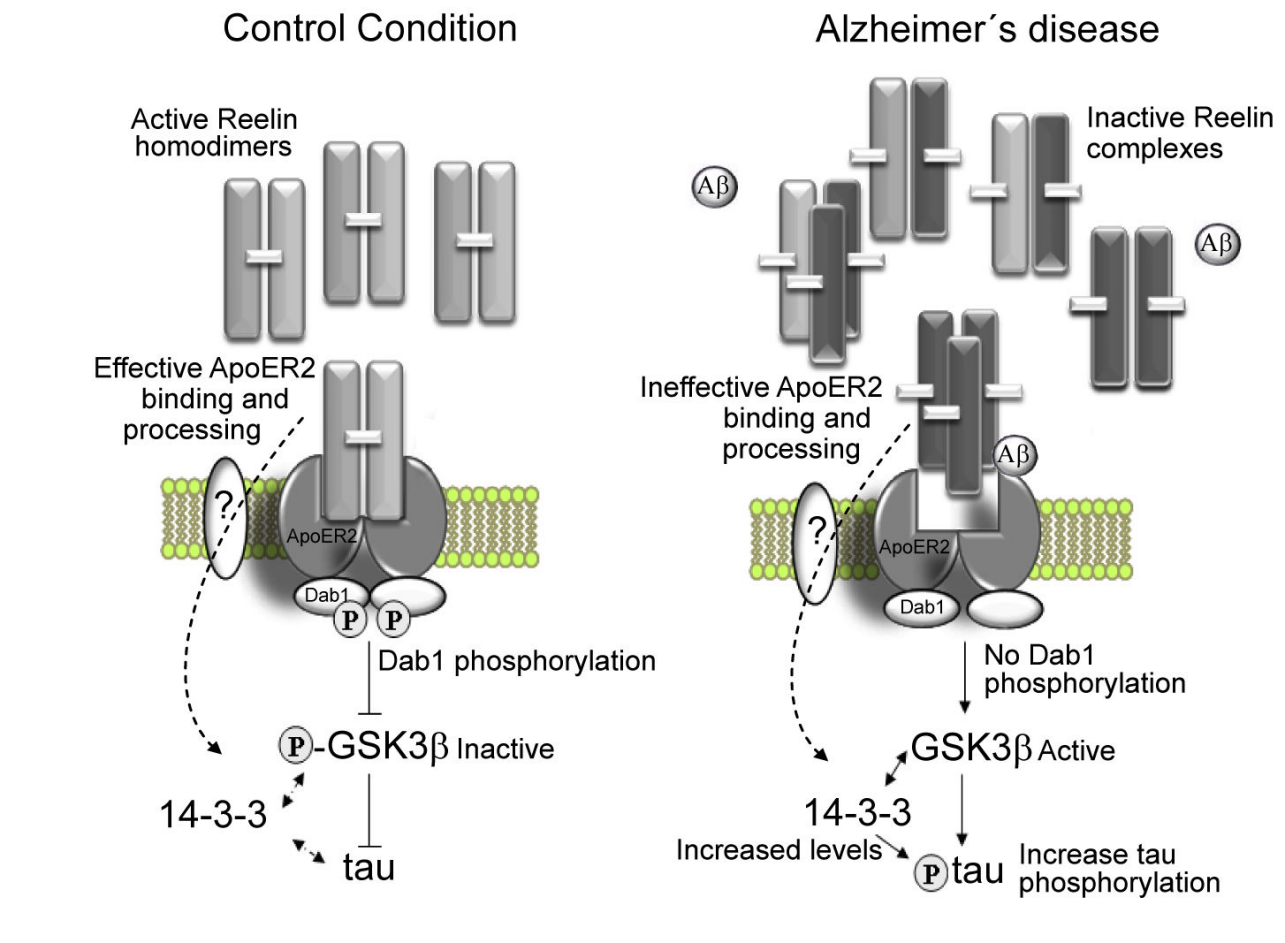
Schematic model of the impaired Reelin signaling in AD. β-Amyloid triggers changes in Reelin glycosylation impairing its ability to form active covalent homodimers. Accordingly, altered Reelin complexes display a reduced efficiency to bind the ApoER2 receptor, fail to induce efficient tyrosine phosphorylation of Dab1, and to transduce the signaling pathway that modulates tau phosphorylation by inhibition of GSK3β. Soluble Aβ can also contribute to the impaired ability of Reelin to activate its signaling cascade. An imbalance in 14-3-3 levels is also triggered by the inefficient Reelin signaling.

Our data indicate that abnormal Reelin species, triggered by Aβ treatment, are inefficient in activating the signaling cascade and in controlling tau phosphorylation. Abnormal Reelin species fail to form active dimers and to bind properly to ApoER2. Interestingly, aberrant Reelin present in the AD brain form large complexes instead of dimers. Thus, abnormal Reelin species may interact with normal forms of the protein, possibly sequestering these to form oligomeric complexes but not physiologically active dimers (Figure 6). Therefore, despite an increase in the total amount of Reelin in the AD brain, less active Reelin may be available. We have also reported in the Tg2576 APP mice increased levels of Reelin [11]. In view of our present results the amount of active (dimers) and inactive (large complexes) Reelin should be analyze in this and others mice models of Aβ over-expression. Furthermore, an increase in abnormal Reelin would result in less activation of the Reelin signaling pathway and could contribute to the generation of a vicious cycle, where Reelin up-regulation may be driven by a chronic failure in Reelin signaling. Up-regulation of proteins in response to chronic inhibition is a recognized phenomenon documented for several proteins [28–30].

Alternative consequences for the lack of modulation of tau phosphorylation by Aβ-Reelin signaling have also been considered. We have demonstrated that neurons treated with Aβ-Reelin display an increase in the scaffold protein 14-3-3, a protein that only when is present maintains GSK3β phosphorylated at serine 9 active and this phosphorylates tau [22]. Whether changes in 14-3-3 levels are part of a compensatory system of the complex tau-GSK3β to regulate GSK3β activity, or directly respond to Reelin signaling through ApoER2 or other receptors remain to be elucidated (Figure 6). Interestingly, it has been shown in platelets that 14-3-3 is translocated to the cytoskeleton after ApoER2 activation [31]. Moreover, Reelin coreceptors, members of the family of cadherin-related neuronal receptors (CNRs) [32] or integrins [33] have been proposed to be involved in the Reelin pathway; however it has also been demonstrated that Reelin binding to ApoER2 itself seems to be sufficient to transmit the signal and does not appear to require any other coreceptor [23]. However, we cannot exclude that such coreceptors participate in other cellular events induced by Reelin binding, including regulation of the 14-3-3 protein. Indeed, 14-3-3 levels can be modulated by integrins [34]. Furthermore, several isoforms of 14-3-3 have been found to be increased in the brains of patients with AD [35], and a recent proteomic approach confirms that 14-3-3 can be a disease-specific protein in AD [36]. Our data indicate that intracellular 14-3-3 levels are similar in cultures treated with either altered glycoforms of Reelin or with Reelin blocked by the CR50 antibody, demonstrating further that altered Reelin glycoforms may sequester active Reelin.

Our previous studies illustrated altered Reelin glycosylation in cells treated with Aβ using immobilized Con A and LCA and a LCA gel-shift assay [11]. Abnormal Reelin species also resemble Reelin detected in the AD brain in altered glycosylation pattern. We have no direct evidence that Aβ mediates changes in Reelin glycosylation compromise Reelin functionality, since it is likely that other cellular components are present in our Reelin-enriched fractions, and particularly Aβ. Similarly, we presume that oligomeric Aβ is the specie that affects the glycosylation of Reelin; however, under the conditions of our cellular experiments, we cannot differentiate if this effect is due to monomeric, oligomeric Aβ or fibers. Moreover, it is possible that Aβ itself adversely affects Reelin signaling (Figure 6). Further studies addressing the possibility that Aβ (monomers or oligomers) interacts directly with Reelin are warranted. Nonetheless, an impaired ability of Reelin to modulate tau phosphorylation was also exhibited by altered Reelin glycoforms induced by DMJ, an inhibitor of mannosidase. Thus, we favor the hypothesis that alterations in Reelin glycosylation affect the ability to form active dimers, bind the receptor and control the extent to which the tau protein is phosphorylated. Discrete changes in the glycosylation profile of key brain proteins could have a critical impact in their function. Indeed, APP (the amyloid-precursor protein) glycosylation can modulate its metabolic turnover [37]. Glycosylation also modulates the function of β-secretase [38] and γ-secretase [39], and the processing of ApoER2 by γ-secretase [40]. In addition, glycosylation has been shown to play a role in modulating the hyperphosphorylation status of tau [41] and the maintenance of paired helical filaments [42]. The physiological relevance of the change in the glycosylation profile for key AD proteins including Reelin is clear; however the mechanism through which Aβ affects the glycosylation of Reelin warrants further study.

In conclusion, we present evidence of the abnormal dimerization of Reelin in the AD brain which suggests an altered signaling *in vivo*. We have shown that Aβ induces expression of abnormal (non physiologically efficient) Reelin species, resulting in impaired Reelin signaling. Our data associates Aβ and tau phosphorylation dysregulation through Reelin and raises the possibility that Reelin directly contributes to the progress of AD pathology.

## Supporting Information

Figure S1 **Aβ42 induces changes in secreted Reelin from cultured cells.**
(**A**) Aβ content was measured in the cell medium from non-treated (NT) or Aβ-treated SH-SY5Y cells before and after preparation procedures, and compared with the amount found in the pellet of the cell medium obtained before filtration. The Aβ was depleted of enriched Reelin fraction by filtration/concentration (filtration) and alternatively by ulterior immunoprecipitation (IP) with anti-Aβ antibody 6E10. There was a neglected amount of the peptide in Reelin enriched concentrates obtained from cell supernatants treated with 1 µM Aβ42 (blot not shown). Positions of the molecular-weight (MW) markers are shown. There was no significant cell death in cultures treated with 10 µM Aβ42, as evaluated by the MTS assay (13 ±5% reduction, *p*= 0.2), and only marginal cell death was estimated in cells treated with 10 µM Aβ42 (28 ±4% reduction, *p*= 0.008). (**B**) The glycosylation status of secreted Reelin from Aβ42-treated SH-SY5Y cells was analyzed by a lectin-binding assay. Comparison of the pattern of unbound Reelin to Con A or LCA are shown from representative cases of Cont-Reelin (C-Reel) and Aβ-Reelin (Aβ-Reel) (representative cases from 4 independent experiments are shown).
(TIF)

Figure S2 **Impaired Reelin-mediated c capacity of Aβ-Reelin.**
Representative photographs of cells attached to a well coated coated without Reelin (No Reel) or with Cont-Reelin (C-Reel), Aβ-Reelin (Aβ-Reel) or Cont-Reelin preincubated with CR50 antibody. The histogram shows the number of cells in each case normalized to Cont-Reelin, which was scored as 100 (n=5 independent experiments, values are means ± SEM). t-Test *p < 0.05, **p < 0.01.
(TIF)
