# Supplementary figures and images for: Beta-Amyloid Impairs Reelin Signaling
Source: PLoS One. 2013 Aug 12;8(8):e72297. doi: 10.1371/journal.pone.0072297 (PMC3741172; doi:10.1371/journal.pone.0072297)

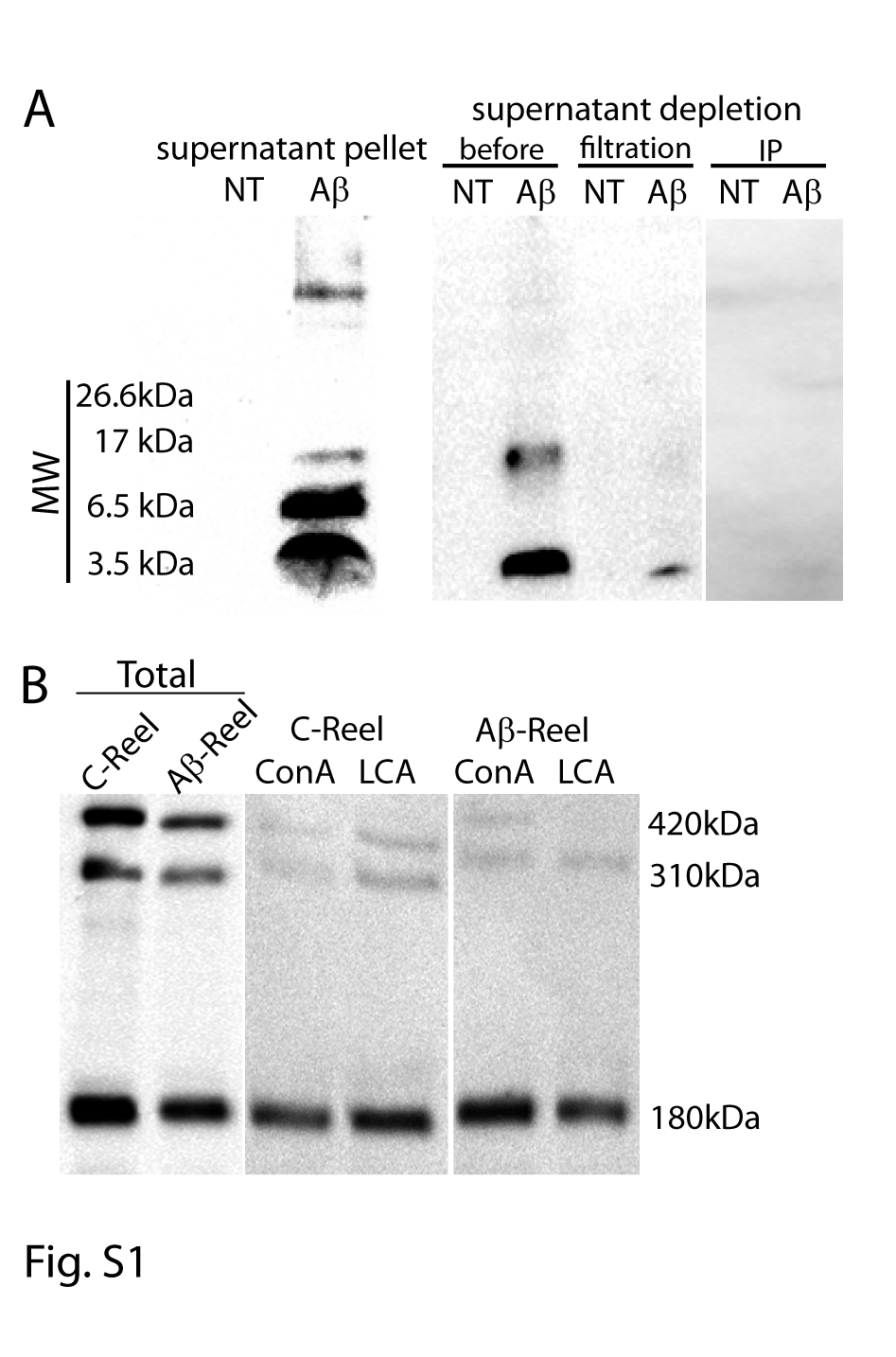

Supplement: Figure S1 — Aβ42 induces changes in secreted Reelin from cultured cells. (A) Aβ content was measured in the cell medium from non-treated (NT) or Aβ-treated SH-SY5Y cells before and after preparation procedures, and compared with the amount found in the pellet of the cell medium obtained before filtration. The Aβ was depleted of enriched Reelin fraction by filtration/concentration (filtration) and alternatively by ulterior immunoprecipitation (IP) with anti-Aβ antibody 6E10. There was a neglected amount of the peptide in Reelin enriched concentrates obtained from cell supernatants treated with 1 µM Aβ42 (blot not shown). Positions of the molecular-weight (MW) markers are shown. There was no significant cell death in cultures treated with 10 µM Aβ42, as evaluated by the MTS assay (13 ±5% reduction, p= 0.2), and only marginal cell death was estimated in cells treated with 10 µM Aβ42 (28 ±4% reduction, p= 0.008). (B) The glycosylation status of secreted Reelin from Aβ42-treated SH-SY5Y cells was analyzed by a lectin-binding assay. Comparison of the pattern of unbound Reelin to Con A or LCA are shown from representative cases of Cont-Reelin (C-Reel) and Aβ-Reelin (Aβ-Reel) (representative cases from 4 independent experiments are shown). (TIF) [file pone.0072297.s001.tif]

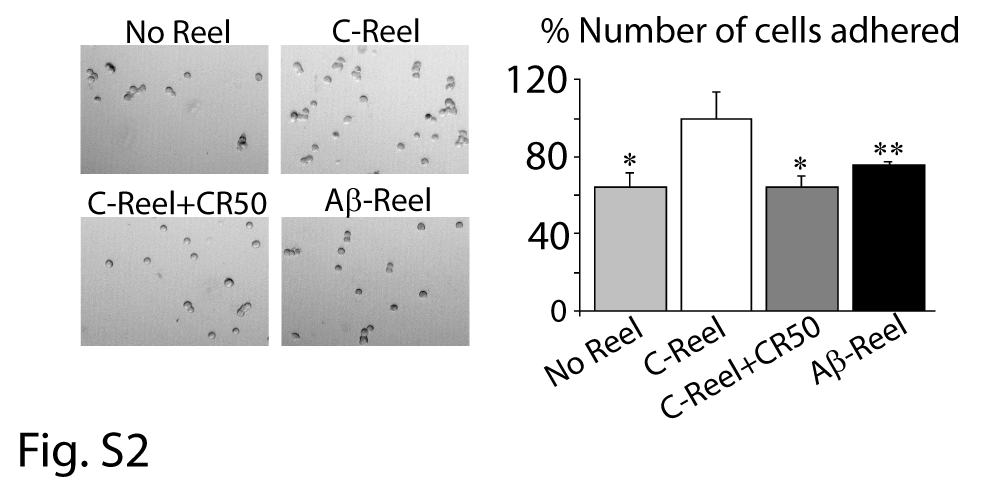

Supplement: Figure S2 — Impaired Reelin-mediated c capacity of Aβ-Reelin. Representative photographs of cells attached to a well coated coated without Reelin (No Reel) or with Cont-Reelin (C-Reel), Aβ-Reelin (Aβ-Reel) or Cont-Reelin preincubated with CR50 antibody. The histogram shows the number of cells in each case normalized to Cont-Reelin, which was scored as 100 (n=5 independent experiments, values are means ± SEM). t-Test *p < 0.05, **p < 0.01. (TIF) [file pone.0072297.s002.tif]
